# Supplementary figures and images for: A dynamic power-law sexual network model of gonorrhoea outbreaks
Source: PLoS Comput Biol. 2019 Mar 8;15(3):e1006748. doi: 10.1371/journal.pcbi.1006748 (PMC6426262; doi:10.1371/journal.pcbi.1006748)

$\gamma = 1.4; k_0 = 0.4; p(k=0) = 0.16$ 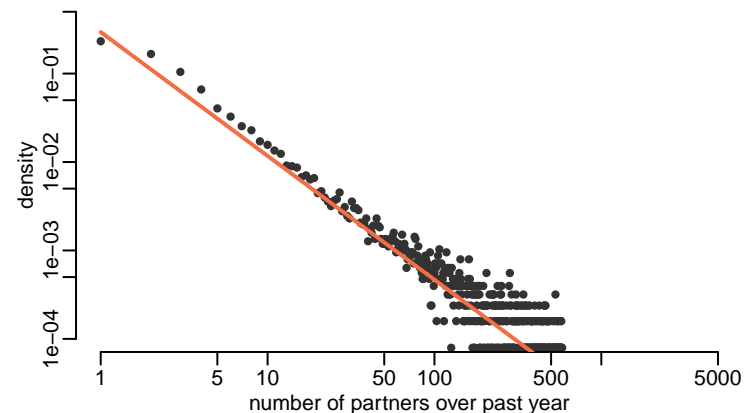 $\gamma = 1.6; k_0 = 0.4; p(k=0) = 0.21$ 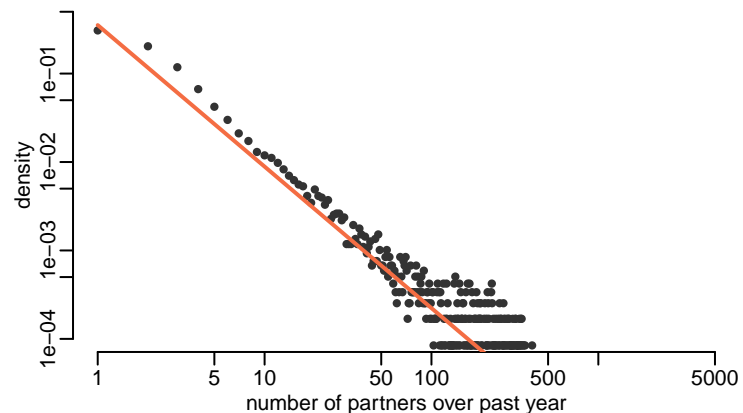 $\gamma = 1.8; k_0 = 0.4; p(k=0) = 0.27$ 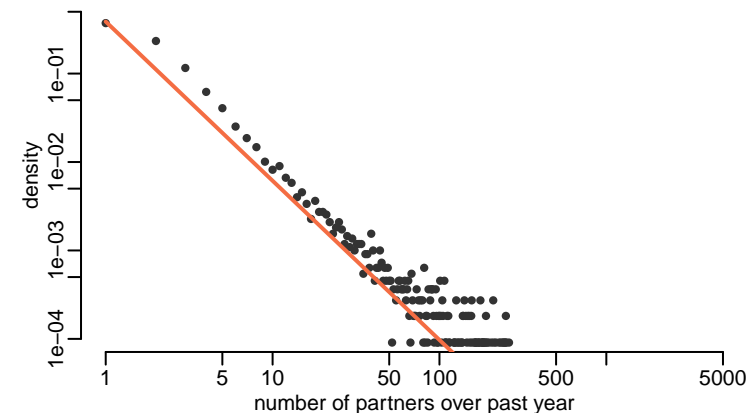 $\gamma = 1.4; k_0 = 0.5; p(k=0) = 0.14$ 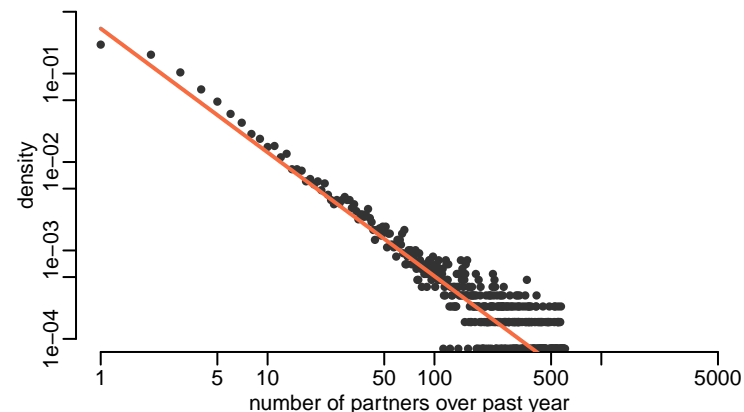 $\gamma = 1.6; k_0 = 0.5; p(k=0) = 0.18$ 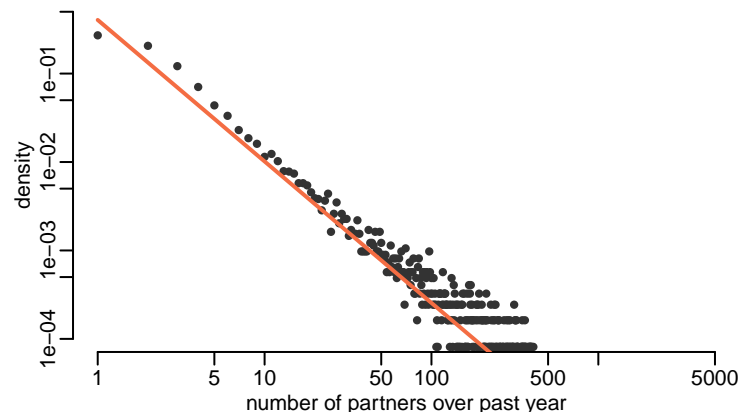 $\gamma = 1.8; k_0 = 0.5; p(k=0) = 0.23$ 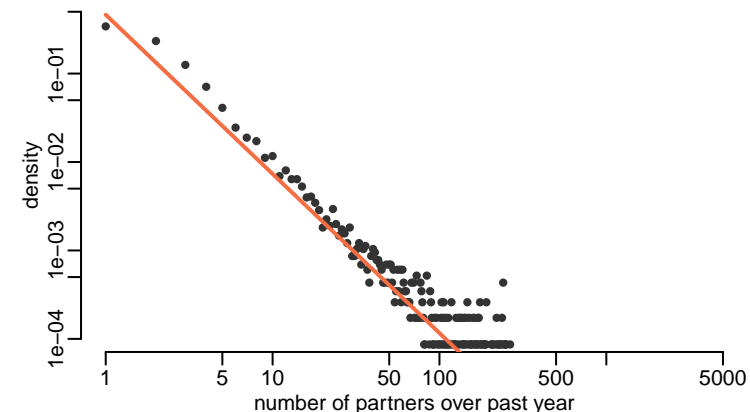 $\gamma = 1.4; k_0 = 0.6; p(k=0) = 0.11$ 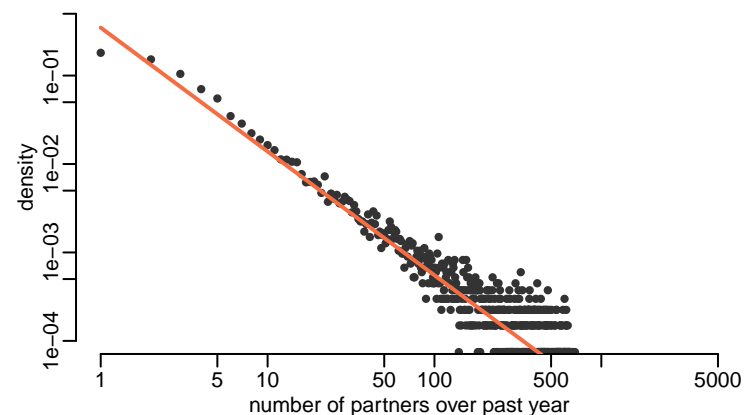 $\gamma = 1.6; k_0 = 0.6; p(k=0) = 0.15$ 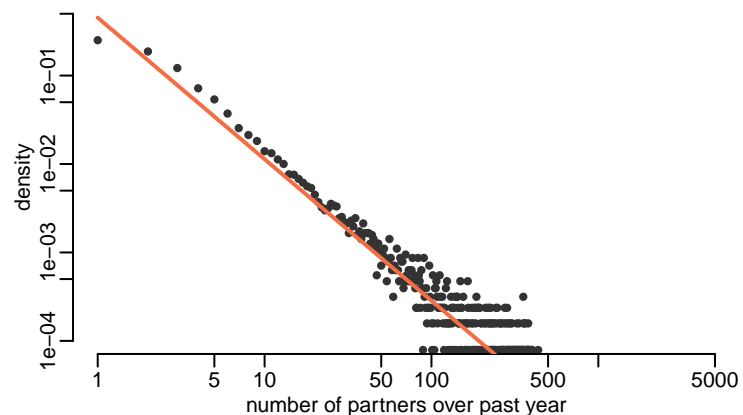
$$\gamma = 1.8; k_0 = 0.6; p(k=0) = 0.2$$
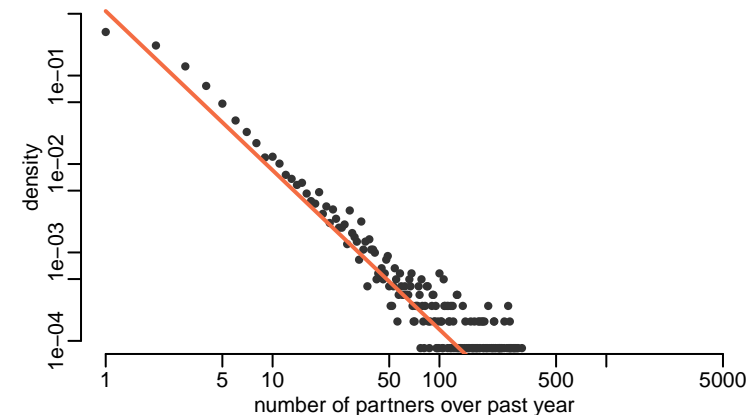

Supplement: S1 Fig — The orange lines represent the desired degree distribution p(k) = ck−γ. The proportion of individuals having no partners in the last year is shown in the title of each plot as p(k = 0). (PDF) [file pcbi.1006748.s001.pdf]

density

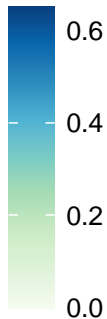

number of siblings

9  
8  
7  
6  
5  
4  
3  
2  
1  
0

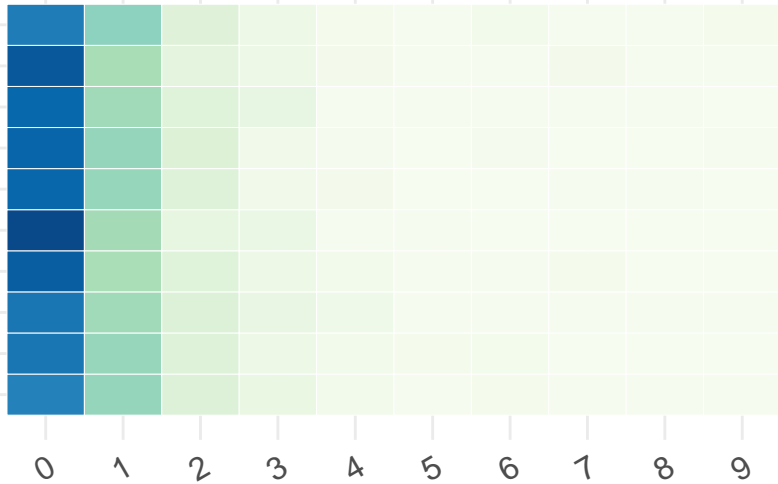

number of offspring

Supplement: S2 Fig — (PDF) [file pcbi.1006748.s002.pdf]

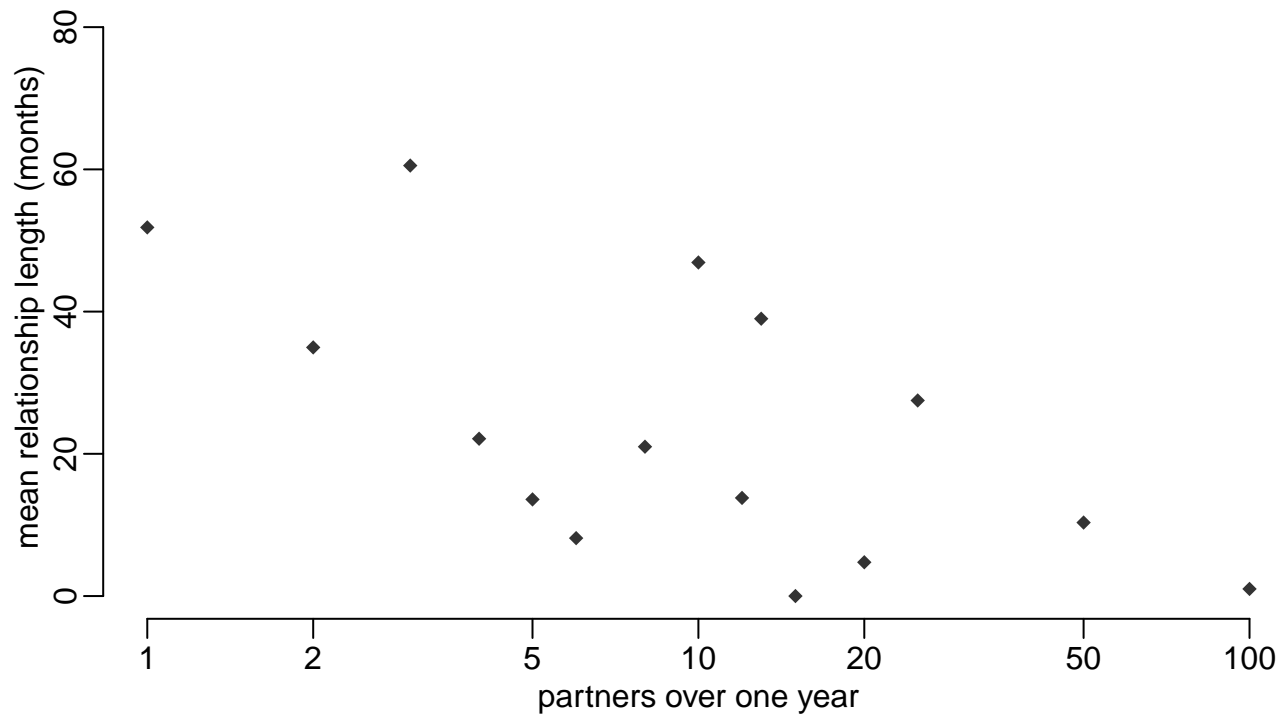

Supplement: S3 Fig — The number of partners reported by MSM over one year is negatively correlated with the average length of the relationships. This suggests that in a realistic model the expected duration of partnerships formed by high degree individuals should be shorter than for low-degree individuals. (PDF) [file pcbi.1006748.s003.pdf]
