# Supplementary material for: A dynamic power-law sexual network model of gonorrhoea outbreaks
Source: PLoS Comput Biol. 2019 Mar 8;15(3):e1006748. doi: 10.1371/journal.pcbi.1006748 (PMC6426262; doi:10.1371/journal.pcbi.1006748)
Supplement: S1 Appendix — (PDF) [file pcbi.1006748.s004.pdf]

S1 Appendix: Simulation of static snapshot of network over one year

We can simulate the static network of partners over the last year very simply using the following algorithm, which takes as inputs: the size of the network N, the distribution of λ, $f(\lambda)$, the desired power-law coefficient γ and $k_0 = \lim_{\lambda \to 0} \kappa(\lambda)$.

1. draw $\lambda_1, \ldots, \lambda_N$ from $f(\lambda)$
2. choose k_∞ to be as large as possible, subject to the condition that $g(\infty) \leq 1$
3. calculate $g(\lambda)$
4. generate an NxN matrix, Q, of the equilibrium probabilities that each possible set of partners $\{j, l\}$ have been in a partnership within the last year where:

   $$q_{j,l} = q(\lambda_j, \lambda_l) = g(\lambda_j)g(\lambda_l)$$

5. generate an NxN matrix U where:

   $$u_{j,l} = \begin{cases} 1 & \text{if } j \geq l \\ \sim U(0,1) & \text{otherwise} \end{cases}$$

   Note that the reason for populating only the lower triangle of the matrix is that the sexual network is undirected, i.e. if $\{j, l\}$ is a pair then $\{l, j\}$ is a pair, so the network matrix will be symmetrical, and therefore can be generated from the lower triangular entries only. The diagonal is not included as it is not possible to form a partnership with oneself.

6. generate an NxN matrix M where:

   $$m_{j,l} = \begin{cases} 1 & \text{if } u_{j,l} < q(\lambda_j, \lambda_l) \\ 0 & \text{otherwise} \end{cases}$$

   The matrix M then represents an undirected network of the partnerships that have existed within the past year.
