# Supplementary material for: A dynamic power-law sexual network model of gonorrhoea outbreaks
Source: PLoS Comput Biol. 2019 Mar 8;15(3):e1006748. doi: 10.1371/journal.pcbi.1006748 (PMC6426262; doi:10.1371/journal.pcbi.1006748)
Supplement: S2 Appendix — (PDF) [file pcbi.1006748.s005.pdf]

S2 Appendix: Simulation of dynamic network

We can simulate the dynamic network of partnerships using the following algorithm, which takes as inputs: the size of the network N, the distribution of λ, $f(\lambda)$, the desired power-law coefficient γ and $k_0 = \lim_{\lambda \to 0} \kappa(\lambda)$.

1. choose k_∞ to be as large as possible, subject to the condition that $g(\infty) \leq 1$
2. draw $\lambda_1, \ldots, \lambda_N$ from $f(\lambda)$
3. simulate partnerships in network at time $t = 0$, where $\mathbb{P}(\{j, l\} \in \mathcal{P}(0)) = q_0(\lambda_j, \lambda_l) = 1 - \frac{1}{1+\frac{1}{\phi}}$
4. calculate $g(\lambda)$
5. generate an NxN matrix of the rates of partnership formation that each possible set of partners A, where:
$$A_{j,l} = \min\left\{ -\ln\left(\left(1 - g(\lambda_j)g(\lambda_l)\right)\left(1 + \frac{1}{\phi}\right)\right), \frac{1}{50\phi}\right\}$$
6. set $A_{j,j} := 0$ for all $j = 1, \ldots, N$
7. determine initial event rates $\hat{R}_a(0), R_d(0)$(*note that* $\hat{R}_a(t) \geq R_a(t)$ *as rejection sampling is used*)
   - rate of adding partnerships $\hat{R}_a(t) = \sum_{j=1}^{N} \sum_{l=j+1}^{N} a(\lambda_j, \lambda_l)$ (time invariant)
   - rate of deleting partnerships $R_d(0) = \sum_{j,l \in \mathcal{P}(0)} d(\lambda_j, \lambda_l)$
8. While $t < T$ where initially $t = 0$:
   (a) Generate a time, $\tau \sim \text{Exp}(\hat{R}(t))$ after which the next event, e takes place;
   (b) update the time
$$t := t + \tau$$
   (c) Determine which event took place by sampling $e \in \{a, d\}$ where
$$\mathbb{P}[e] = \frac{\hat{R}_e(t-\tau)}{\hat{R}(t-\tau)}$$
   - if the event sampled is a partnership formation ($e = a$):
     i. propose two individuals $\{j, l\}$ from the population with probability $\frac{a(\lambda_j, \lambda_l)}{\hat{R}_a(t-\tau)}$.
     ii. check the partnership does not already exist, if $\{j, l\} \in \mathcal{P}(t-\tau)$ reject an move on to next step;
     iii. else, accept and update the state of the model
$$\mathcal{P}(t) := \mathcal{P}(t-\tau) \cup \{j, l\}$$
$$N_P(t) := N_P(t-\tau) + 1$$
     iv. increase rate of partnership breakups
$$R_d(t) := R_d(t-\tau) + d(\lambda_j, \lambda_l)$$
   - if the event sampled is a partnership breakup ($e = d$):
     i. sample a partnership to breakup $\{j, l\}$ from the current couples $\mathcal{P}(t-\tau)$ with probability $\frac{d(\lambda_j, \lambda_l)}{R_d(t-\tau)}$

ii. update the state of the model

$$\mathcal{P}(t) \coloneqq \mathcal{P}(t-\tau) \setminus \{j, l\}$$

$$N_P(t) \coloneqq N_P(t-\tau) - 1$$

iii. decrease rate of partnership breakups

$$R_d(t) \coloneqq R_d(t-\tau) - d(\lambda_j, \lambda_l)$$

(d) next step

9. return the states of the model $\mathcal{P}(t)$ for all sampled times t

At any given time we have

- $\hat{R}_a(t) = \sum_{j=1}^{N} \sum_{l=j+1}^{N} a(\lambda_j, \lambda_l)$;
- $R_d(t) = \sum_{\{j,l\} \in \mathcal{P}(t-\tau)} d(\lambda_j, \lambda_l)$;
