# Supplementary material for: A dynamic power-law sexual network model of gonorrhoea outbreaks
Source: PLoS Comput Biol. 2019 Mar 8;15(3):e1006748. doi: 10.1371/journal.pcbi.1006748 (PMC6426262; doi:10.1371/journal.pcbi.1006748)
Supplement: S3 Appendix — (PDF) [file pcbi.1006748.s006.pdf]

S3 Appendix: Derivation of parameter input ranges

Proportion of new infections that develop symptoms - ψ

The proportion of incident infections that are symptomatic is known to vary by site of infection. Traditional modelling attempts using the few empirical studies available estimated the proportion of urethral cases that develop symptoms at 90% to 95% [1–3]. Extra-genital infections are less likely to develop symptoms. In screening studies on MSM attending a sexual health clinics only 15% of rectal and none of the pharyngeal infections detected were symptomatic [4–6]. We therefore consider scenarios in which 40% to 95% of new infections develop symptoms, to reflect scenarios in which 50%-100% of infections are urethral.

Mean duration of incubation period - $1/\sigma$

The incubation period of gonorrhoea, defined as the time from initial infection to exhibiting symptoms of disease, was traditionally thought to be short - in the region of three to five days [2, 7, 8]. It has been suggested that the incubation period may be increasing, perhaps as a result of antibiotics usage, to around 8 days [9, 10]. We therefore considered a range of incubation periods from 2 to 10 days in our simulations.

Mean duration of carriage - $1/\nu$

There is little data on the duration of untreated gonorrhoea, which may depend on infection site. Recovery time for pharyngeal gonorrhoea is estimated at 12 weeks [11, 12]. By contrast, the duration of rectal infection has been estimated at one year [11, 13]. A study of 18 asymptomatic infected men saw no resolution in urethral infection in the 165 days until they received treatment [3]. Estimates of the duration of asymptomatic infection in modelling studies have been based on calculations that take into account observed prevalence and assumed proportion of unobserved infection, and are often in the region of 6 months [1, 14, 15]. This was supported by evidence based on genomic data in which the longest observed time to most recent common ancestor was 8 months [16]. Researchers using high sensitivity diagnostic tools in Namibia found evidence of a much longer duration of carirage. They estimated a 333-day average term of high-level infection before progression to low-level infection [17]. Low-level urethral infections took an average 250 days to resolve without treatment. We therefore considered a range of carriage durations from 2 - 18 months.

Rate of screening - η

The 2010 UK national guidelines on safer sex advice recommend that all MSM are screened for gonorrhoea at least annually, and that highly sexually active MSM are screened at least every three months [18, 19]. However a social media survey of 2,668 MSM in Scotland, Wales and Ireland found that two-thirds of MSM reported STI testing less than once a year [20]. We considered a range of inputs for the rate of screening of [0.4, 4]. This corresponds to a range of scenarios: from testing at quarterly intervals, in adherence with the BASSH guidelines, through to the situation described by Frankis et al. The lower bound for eta was calculated by setting the probability of testing less than once a year equal to $\frac{2}{3}$ under an exponential distribution with rate η, so that $\mathcal{P}[T > 1] = e^{-\eta} = \frac{2}{3}$, and solving to find $\eta = -\ln \frac{2}{3} = 0.4$.

The mean time to treatment seeking when symptomatic $(1/\mu)$ and mean time to recovery following treatment $(1/\rho)$ were set to be in the region of days rather than weeks.

References

1. Hethcote HW, Yorke JA. Modeling Gonorrhea in a Population with a Core Group; 1984. p. 32–48. Available from: `http://link.springer.com/10.1007/978-3-662-07544-9{_}4`.

2. Harrison WO, Hooper RR, Wiesner PJ, Campbell AF, Karney WW, Reynolds GH, et al. A trial of minocycline given after exposure to prevent gonorrhea. New England Journal of Medicine. 1979;300(19):1074–1078. doi:10.1056/NEJM197905103001903.

3. Handsfield HH, Lipman TO, Harnisch JP, Tronca E, Holmes KK. Asymptomatic Gonorrhea in Men. New England Journal of Medicine. 1974;290(3):117–123.

4. Bissessor M, Tabrizi SN, Fairley CK, Danielewski J, Whitton B, Bird S, et al. Differing Neisseria gonorrhoeae bacterial loads in the pharynx and rectum in men who have sex with men: Implications for gonococcal detection, transmission, and control. Journal of Clinical Microbiology. 2011;49(12):4304–4306. doi:10.1128/JCM.05341-11.

5. Kent CK, Chaw JK, Wonq W, Liska S, Gibson S, Hubbard G, et al. Prevalence of rectal, urethral, and pharyngeal chlamydia and gonorrhea detected in 2 clinical settings among men who have sex with men: San Francisco, California, 2003. Clinical Infectious Diseases. 2005;41(1):67–74. doi:10.1086/430704.

6. Norris Turner A, Carr Reese P, Ervin M, Davis JA, Fields KS, Bazan JA. HIV, rectal chlamydia and rectal gonorrhea in men who have sex with men attending an STD clinic in a midwestern US city. Sexually Transmitted Diseases. 2013;40(6). doi:10.1097/OLQ.0b013e31828fd163.HIV.

7. McCutchan JA. Epidemiology of venereal urethritis: comparison of gonorrhea and nongonococcal urethritis. Reviews of Infectious Diseases. 1984;6(5):669–688.

8. Korenromp EL, Sudaryo MK, de Vlas SJ, Gray RH, Sewankambo NK, Serwadda D, et al. What proportion of episodes of gonorrhoea and chlamydia becomes symptomatic? International Journal of STD and AIDS. 2002;13(2):91–101. doi:10.1258/0956462021924712.

9. LODIN A. Has the incubation period of gonorrhoea undergone a change? Acta dermato-venereologica. 1955;35(6):457–462.

10. Sherrard J, Barlow D. Gonorrhoea in men: clinical and diagnostic aspects. Genitourinary medicine. 1996;72(6):422–6. doi:10.1136/sti.72.6.422.

11. Fairley CK, Chen MY, Bradshaw CS, Tabrizi SN. Is it time to move to nucleic acid amplification tests screening for pharyngeal and rectal gonorrhoea in men who have sex with men to improve gonorrhoea control? Sexual Health. 2011;8(1):9–11. doi:10.1071/SH10134.

12. Wallin J, Siegel, M S. Pharyngeal neisseria gonorrhoeae: coloniser or pathogen? British Medical Journal. 1979;1(6176):1462–1463.

13. Jin F, Prestage GP, Mao L, Kippax SC, Pell CM, Donovan B, et al. Incidence and risk factors for urethral and anal gonorrhoea and chlamydia in a cohort of HIV-negative homosexual men: the Health in Men Study. Sexually Transmitted Infections. 2007;83(2):113–119. doi:10.1136/sti.2006.021915.

14. White PJ, Ward H, Cassell JA, Mercer CH, Garnett GP. Vicious and virtuous circles in the dynamics of infectious disease and the provision of health care: gonorrhea in Britain as an example. The Journal of Infectious Diseases. 2005;192(5):824–836. doi:10.1086/432004.

15. Johnson LF, Geffen N. A Comparison of Two Mathematical Modeling Frameworks for Evaluating Sexually Transmitted Infection Epidemiology. Sexually transmitted diseases. 2016;43(3):139–46. doi:10.1097/OLQ.0000000000000412.

16. Didelot X, Dordel J, Whittles LK, Collins C, Bilek N, Bishop CJ, et al. Genomic analysis and comparison of two gonorrhoea outbreaks. mBio. 2016;7(3).

17. Hazel A, Marino S, Simon C. An anthropologically based model of the impact of asymptomatic cases on the spread of {{}Neisseria{}} gonorrhoeae. Journal Of The Royal Society Interface. 2015;12(106):20150067. doi:10.1098/rsif.2015.0067.

18. Clutterbuck D, Flowers P, Barber T, Wilson H, Nelson M, Hedge B, et al. The 2010 UK national guidelines on safer sex advice. The Clinical Effectiveness Group of the British Association for Sexual Health and HIV (BASHH) and the British HIV Association (BHIVA); 2010. January. Available from: http://www.bhiva.org/documents/Guidelines/SaferSex/Guideline{_}on{_}Safer{_}Sex{_}Advice.pdf.

19. Ross J, Brady M, Clutterbuck D, Doyle T, Hart G, Hughes G, et al. BASHH Recommendations for Testing for Sexually Transmitted infections in Men who have Sex with Men. British Association of Sexual Health and HIV; 2014. Available from: http://www.bashh.org/documents/BASHHRecommendationsfortestingforSTIsinMSM-FINAL.pdf.

20. Frankis J, Goodall L, Clutterbuck D, Abubakari R, Flowers P. Regular STI testing amongst men who have sex with men and use social media is suboptimal – a cross-sectional study. International Journal of STD & AIDS. 2017;28(6):573–583. doi:10.1177/0956462416636780.
